# Re: predictive modeling of treatment-related thyroid dysfunction and prognostic implication in advanced nasopharyngeal carcinoma with PD-1 inhibitors

**DOI:** 10.1093/oncolo/oyag188

**Published:** 2026-05-16

**Authors:** Polina Shilo

**Affiliations:** Lahta Clinic Medical Center, St. Petersburg, 197374, Russia

Hu et al. address an important clinical question by examining whether treatment-related thyroid dysfunction (trTD) during PD-1 therapy may also have prognostic significance in advanced nasopharyngeal carcinoma. The study is of interest, particularly because it describes both the frequency of trTD and the temporal pattern of thyroid function changes during treatment.

However, the survival analyses require careful interpretation. trTD is not a baseline characteristic but an event that develops during follow-up. In this study, progression-free and overall survival were calculated from the first dose of PD-1 therapy, whereas patients were compared according to whether trTD subsequently occurred. At the same time, the authors show that thyroid changes accumulated over the course of treatment, with TSH rising after approximately the fourth to fifth cycle and trTD occurring more frequently among patients who received more than 5 cycles. Under these conditions, patients had to remain on treatment and progression-free long enough to develop trTD and to be classified accordingly. This raises concern for immortal time bias if trTD is analyzed as a fixed exposure from time zero rather than as a time-varying variable.

A second issue relates to the interpretation of the survival results. In the Results section, trTD is reported as an independent predictor of improved progression-free survival. For overall survival, however, the multivariable analysis does not appear to provide equivalent support. The language used in the Discussion and Conclusion is therefore somewhat broader than the level of evidence shown in the adjusted analyses, particularly when referring to improved survival more generally.

These considerations do not lessen the clinical relevance of thyroid monitoring during PD-1 therapy. They do suggest that the prognostic significance of trTD would be more appropriately assessed in a time-dependent survival model and that the interpretation should remain closely aligned with the differential strength of evidence for progression-free and overall survival.
